# Protection Elicited by Nasal Immunization with Recombinant Pneumococcal Surface Protein A (rPspA) Adjuvanted with Whole-Cell Pertussis Vaccine (wP) against Co-Colonization of Mice with *Streptococcus pneumoniae*

**DOI:** 10.1371/journal.pone.0170157

**Published:** 2017-01-19

**Authors:** Rafaella O. Tostes, Tasson C. Rodrigues, Josefa B. da Silva, Alessandra S. Schanoski, Maria Leonor S. Oliveira, Eliane N. Miyaji

**Affiliations:** Laboratório de Bacteriologia, Instituto Butantan, São Paulo, SP, Brazil; Universidad Nacional de la Plata, ARGENTINA

## Abstract

A promising alternative vaccine candidate to reduce the burden of pneumococcal diseases is the protein antigen PspA (Pneumococcal surface protein A). Since concomitant colonization with two or more pneumococcal strains is very common in children, we aimed to determine if immunization with PspA would be able to control co-colonization. We evaluated nasal immunization with recombinant PspA (rPspA) in a model of co-colonization with two strains expressing different PspAs. Mice were immunized intranasally with rPspAs from clades 1 to 4 (rPspA1, rPspA2, rPspA3 or rPspA4) using whole-cell pertussis vaccine (wP) as adjuvant. Mice were then challenged with a mixture of two serotype 6B isolates St491/00 (PspA1) and St472/96 (PspA4). Immunization with rPspA1+wP and rPspA4+wP reduced colonization with both strains and the mixture of rPspA1+rPspA4+wP induced greater reduction than a single antigen. Immunization rPspA1+rPspA4+wP also reduced colonization when challenge experiments were performed with a mixture of isolates of serotypes 6B (PspA3) and 23F (PspA2). Furthermore, none of the tested formulations led to a pronounced increase in colonization of one isolate over the other, showing that the vaccine strategy would not favor replacement. Interestingly, the adjuvant wP by itself already led to some reduction in pneumococcal colonization, indicating the induction of non-specific immune responses. Anti-rPspA IgG was observed in serum, nasal wash (NW) and bronchoalveolar lavage fluid (BALF) samples, whereas animals inoculated with formulations containing the adjuvant wP (with or without rPspA) showed higher levels of IL-6 and KC in NW and increase in tissue macrophages, B cells and CD4^+^T cells in BALF.

## Introduction

*Streptococcus pneumoniae* is part of the nasopharyngeal microbiota of healthy humans, maintaining a commensal relationship with the host. However, it can cause several diseases with high mortality and morbidity, such as meningitis, bacteremia and pneumonia, and other common respiratory tract infections such as otitis media and sinusitis. Colonization of the nasopharynx is a prerequisite for pneumococcal disease development and transmission of bacteria. Colonization rates vary according to geographical location and socioeconomic conditions, with prevailing rates of 20–90% in children less than five years of age, and 1–10% in adults [1–4]. Simultaneous colonization by multiple pneumococcal strains is also common and up to 50% of colonized children carry simultaneously two or more strains of *S*. *pneumoniae* [5–8].

The currently available vaccines are based on the response against the capsular polysaccharide (PS) and PS-conjugate vaccines (PCVs) show high efficacy against invasive pneumococcal disease (IPD) caused by vaccine serotypes. Though a net reduction in pneumococcal disease was observed in most countries, the widespread use of PCVs led to the substitution of prevalent serotypes, with an increase in incidence of non-vaccine serotype strains both in colonization [2,9] and IPD [10–13]. This phenomenon of serotype replacement is an example of the effect of the selective pressure promoted by vaccines that do not have complete coverage against all variants of the pathogen.

Protein antigens are being studied as alternative vaccines against pneumococcal disease and a promising candidate is Pneumococcal surface protein A (PspA). Mature PspA is composed of a domain at the C-terminal region that anchors the protein to the cell surface through interaction with choline residues of teichoic and lipoteichoic acids. This domain is followed by a central proline-rich domain and an N-terminal α-helical component exposed on the bacterial surface [14]. PspA shows variability in different isolates and sequence-based classification divide PspA variants into three families, that are further subdivided into six clades: family 1 (clades 1 and 2), family 2 (clades 3, 4 and 5) and family 3 (clade 6) [15]. To achieve complete coverage, it was suggested that a PspA-based vaccine should contain at least one PspA from each of the two major families (1 and 2) [16]. Our group has previously shown that parenteral immunization of mice with a recombinant PspA from clade 4 (rPspA4, family 2) or from clade 5 (rPspA5, family 2) induces protection against lethal pneumococcal challenge with strains expressing PspA from families 1 and 2 [17]. Furthermore, the use of the whole-cell pertussis vaccine (wP) as adjuvant for nasal immunization of mice with rPspA5 induced protection against a lethal intranasal challenge model and also against nasal colonization with a pneumococcal strain expressing PspA from family 1.

Studies that analyzed the genomes of 240 pneumococcal strains from a multidrug resistant lineage [18] and the genomes of 616 strains isolated from the nasopharynx from healthy individuals after the introduction of the heptavalent conjugate pneumococcal vaccine (PCV7) [19] showed that besides loci encoding a putative genetic element and the loci involved in capsule synthesis, the genes under selective pressure with the highest recombination rates were *pspA* and *pspC*. It was thus proposed that these surface antigens are important and involved in the evasion of the host immune system, but their use as vaccines could lead to a phenomenon similar to the serotype replacement seen with the use of PCVs. Thus, the new work reported herein aimed to evaluate the selective pressure of the immunization with rPspA in a co-colonization model of mice, mimicking the natural situation of colonization of the nasopharynx with multiple strains. Mice were immunized intranasally with different rPspAs using wP as adjuvant and two co-colonization models were evaluated. The first co-colonization model uses two serotype 6B strains, one expressing PspA from family 1 and another expressing PspA from family 2. The second model uses one serotype 23F strain expressing PspA from family 1 and one serotype 6B strain expressing PspA from family 2. This model thus aims to analyze the effectiveness of vaccination with rPspA upon exposure to different pneumococci, a common situation, especially in children.

## Material and Methods

### Ethics Statement

This study was performed according to the guidelines outlined by the Brazilian National Council for Control of Animal Experimentation (CONCEA). Experimental protocols were approved by the Ethic Committee on Animal Use of the Butantan Institute (CEUAIB) under protocol number 1158/13. Animals were housed under controlled temperature and light cycle (12/12 hours, light/dark cycle) with daily monitoring. Food and water were given ad libitum.

### Bacterial Strains and Growth Conditions

*S*. *pneumoniae* strains were grown in Todd-Hewitt broth (Difco) supplemented with 0.5% yeast extract (THY) at 37°C without shaking. Bacteria were always plated in blood agar and grown overnight at 37°C before inoculation in THY. Stocks were maintained at −80°C in THY containing 20% glycerol. Strains St491/00 (serotype 6B, PspA1) and St472/96 (serotype 6B, PspA4, resistant to trimethoprim) were kindly provided by Dr Maria Cristina Brandileone (Instituto Adolfo Lutz, São Paulo, Brazil). Strains SPEC 6B (6B OPKA—serotype 6B, PspA3, resistant to spectinomycin) and EMC 23F (23F OPKA–serotype 23F, PspA 2) were kindly provided by Dr Moon Nahm (University of Alabama at Birmingham, USA).

### Recombinant Proteins and Vaccine Formulations

The N-terminal fragments of PspA from clades 1, 2, 3 and 4 (from strains St 435/96, St371/00, St259/98 and St255/00, respectively) were expressed in *Escherichia coli* BL21 SI (Invitrogen) and purified by metal affinity chromatography as previously described [20]. For immunization experiments, recombinant proteins (rPspA1, rPspA2, rPspA3 and rPsp4) were treated with Triton X-114 for removal of LPS [21]. The whole cell pertussis vaccine (wP) used in this work is composed of the whole bacteria inactivated with 0.2% formalin and is currently produced by Instituto Butantan (São Paulo, Brazil).

### Immunization of Mice

Five- to 7-week-old female specific-pathogen-free C57BL/6 mice were obtained from the Medical School of the University of São Paulo (FM-USP, São Paulo, Brazil). Proteins were given through the nasal route in saline. Groups of 5 to 6 animals were given two doses of 5 μg of protein containing 1/8 of the human dose of wP with a 14-day interval. 1/8 of the human dose of wP is the highest dilution used in mice in the potency tests of pertussis vaccine. For immunization with a mixture of two proteins, 2.5 μg of each protein was inoculated. Animals injected only with saline or only with wP were used as controls. Vaccines were administered in a volume of 10 μl using a micropipette. Nasal immunization was conducted in mice previously anesthetized through the intraperitoneal (ip) route with 200 μl of a 0.2% xylazine and 0.5% ketamine mixture. Serum was collected two weeks after the second immunization for the evaluation of antibody levels. Bronchoalveolar lavage fluid (BALF) and nasal wash (NW) samples were collected, as previously described [22], 3 weeks after the second immunization (D0) or at days 1 (D1) and 5 (D5) after co-colonization challenge.

### Measurement of Antibodies by Enzyme-Linked Immunosorbent Assay (ELISA) in Serum Samples

ELISA was carried out as described previously [17] in plates coated with 1 μg/ml rPspA. For the detection of serum antibodies, goat anti-mouse IgG conjugated with horseradish peroxidase (Sigma-Aldrich) was used as secondary antibody. The titer was defined as the reciprocal of the highest dilution with an *A*_492_ of 0.1.

### Co-colonization Challenge

Twenty one days after the last immunization, mice were anesthetized through the ip route with 200 μl of a 0.2% xylazine and 0.5% ketamine solution and a mixture containing two strains of pneumococci (5x10^5^ CFU each) was inoculated intranasally in a volume of 10 μl into both nostrils. Mice were euthanized through the ip route with a lethal dose of a xylazine/ketamine solution (60 mg/g and 300 mg/g, respectively) 5 days later. Animals did not show any sign of sickness after challenge. Pneumococcal loads were determined in nasal washes by plating serial dilutions of the samples on blood agar, as previously described [22]. For challenge with the mixture St491/00 and St472/96, samples were platted on blood agar without antibiotics or with 25 μg/ml trimethoprim. For challenge with the mixture 6B OPKA and 23F OPKA, samples were plated on agar plates without antibiotics or with 300 μg/ml spectinomycin.

### Measurement of Cytokines/Chemokines in BALF and NW Samples

A Luminex-based assay (Milliplex Map Mouse Cytokine/Chemokine Magnetic Bead Panel—Merck Millipore) was used to measure IFN-γ, IL-1-β, IL-5, IL-6, IL-10, IL17A, KC/CXCL1, MCP-1/CCL-2, MIP-2/CXCL2 and TNF-α in BALF and NW samples.

### Immunophenotyping of BALF Cells

BALF cells were immunophenotyped with anti-F4/80 PE-Cy7, anti-CD11c APC, anti-CD11b BB515, anti-CD4 APC-Cy7, anti-CD8 PerCP-Cy5.5, anti-B220 PE, anti-Ly6G BV421 using FACSCanto II (BD Biosciences). Analysis was performed using FlowJo V10 software.

### Statistical Analysis

Statistical analysis was performed using GraphPad Prism 5.0 (GraphPad). Differences were determined using One-way Analysis of Variance (ANOVA) with Tukey’s Multicomparison Test.

## Results

### Induction of Anti-rPspA Antibodies by Immunization with rPspA+wP

**Fig 1:**
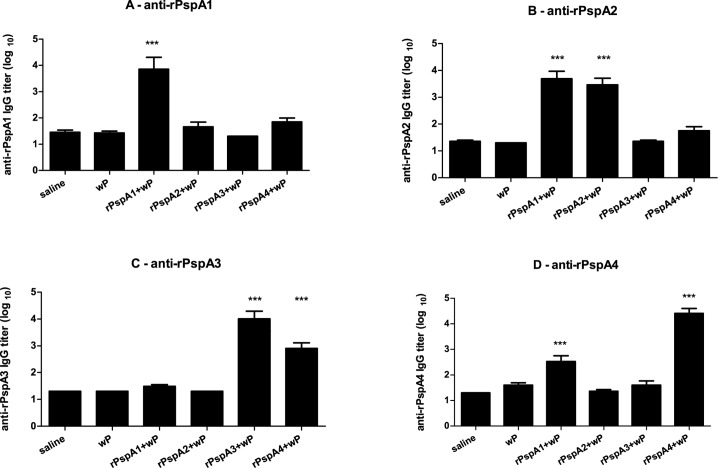
Induction of anti-rPspA IgG serum antibodies. Mice were immunized intranasally with two doses of the indicated formulations and anti-rPspA1(A), anti-rPspA2 (B), anti-rPspA3 (C) and anti-rPspA4 (D) IgG in serum samples were detected by ELISA. * indicates statistical difference with saline (One-way ANOVA, Tukey’s Multicomparison Test—*** P≤0.001). Results are representative of two independent experiments.

C57BL/6 mice were immunized twice intranasally with rPspA1, rPspA2, rPspA3 or rPspA4 using whole-cell pertussis vaccine (wP) as adjuvant. Anti-rPspA IgG levels were determined in serum samples collected two weeks after the last immunization. Mice immunized with rPspA1+wP showed a statistically significant increase in anti-rPspA1, anti-rPspA2 and anti-rPspA4 IgG titers, whereas animals immunized with rPspA4+wP showed a statistically significant increase in anti-rPspA3 and anti-rPspA4 IgG antibodies. Groups immunized with rPspA2+wP and rPspA3+wP showed increase only in antibodies against rPspA2 and rPspA3, respectively (Fig 1). Serum IgG antibodies raised against rPspA1+wP were able to bind to the surface of intact bacteria expressing PspA1 or PspA2 (both family 1), whereas antibodies raised against rPspA4+wP were able to bind to the surface of intact bacteria expressing PspA3 or PspA4 (both family 2) (S1 Fig). These results indicate that rPspA1 (family 1) and rPspA4 (family 2) would be the antigens with broader coverage, at least within the same family.

### Co-colonization Challenge Using Serotype 6B Strains Expressing Different PspAs

**Fig 2:**
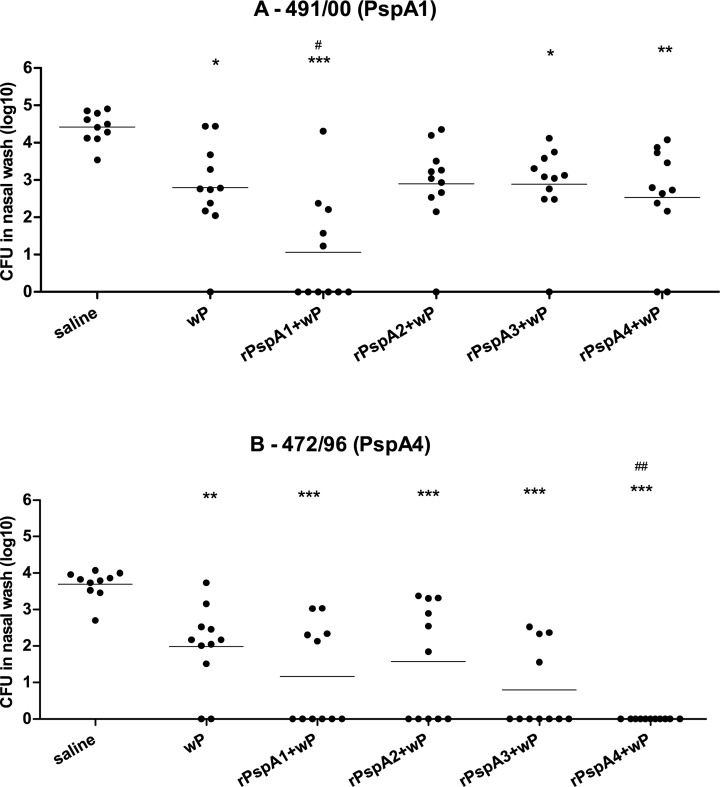
Recovery of pneumococci from the nasopharynx of mice challenged with a mixture of two 6B strains. Mice were immunized intranasally with two doses of the indicated formulations and challenged with a mixture of two 6B strains expressing different PspAs. Recovery of strains St491/00 (PspA1) (A) and St472/96 (PspA4) (B) in nasal washes performed 5 days after challenge is shown. * and ^#^ indicate statistical difference with saline and wP, respectively (One-way ANOVA, Tukey’s Multicomparison Test—* or ^#^ P≤0.05; ** P≤0.01; *** P≤0.001). Results are from two independent experiments.

Mice immunized with the different rPspAs plus wP were challenged intranasally with a mixture of two 6B strains expressing PspAs from family 1 or family 2: St491/00 (PspA1) and St472/96 (PspA4). Bacteria were recovered from nasal washes 5 days after challenge. Animals immunized with wP, rPspA1+wP, rPspA3+wP and rPspA4+wP showed statistically significant reduction in colonization with the two isolates when compared to the control group saline. Mice immunized with rPspA1+wP also showed a statistically significant reduction in colonization with St491/00 (PspA1) when compared to the adjuvant group wP, whereas mice immunized with rPspA4+wP showed statistically significant reduction in colonization with St472/96 (PspA4) when compared to the adjuvant group wP (Fig 2).

**Fig 3:**
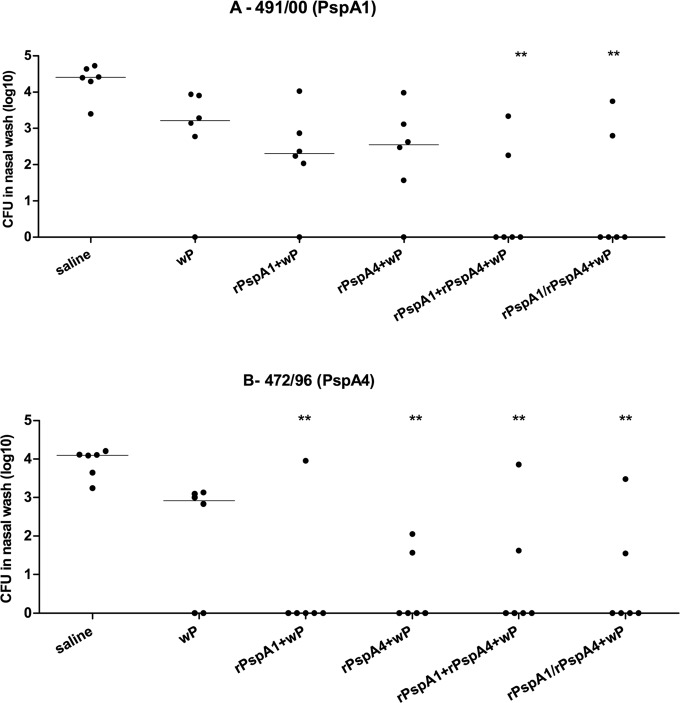
Recovery of pneumococci from the nasopharynx of mice challenged with a mixture of two 6B strains–rPspA1 and rPspA4 mixture. Mice were immunized intranasally with two doses of the indicated formulations and challenged with a mixture of two 6B strains expressing different PspAs. Recovery of strains St491/00 (PspA1) (A) and St472/96 (PspA4) (B) in nasal washes performed 5 days after challenge is shown. * indicates statistical difference with saline (One-way ANOVA, Tukey’s Multicomparison Test—** P≤0.01).

From the above data, rPspA1+wP and rPspA4+wP formulations appear to have a greater potential for protection against St491/00 (PspA1) and St472/96 (PspA4). Thus, the mixture of the two antigens (rPspA1+rPspA4+wP) or a first dose with rPspA1+wP followed by a second dose with rPspA4+wP (rPspA1/rPspA4+wP) were tested to assess the efficacy of these formulations in reducing the colonization by the two strains of pneumococcus. Immunization with either rPspA1+rPspA4+wP or rPspA1/rPspA4+wP led to a greater reduction of colonization by both strains when compared to immunization with a single antigen. Statistically significant reduction of both St491/00 (PspA1) and St472/96 (PspA4) was observed for rPspA1+rPspA4+wP and rPspA1/rPspA4+wP when compared to saline, but not with the adjuvant group wP (Fig 3).

### Co-colonization Challenge Using Serotype 23F and Serotype 6B Strains Expressing Different PspAs

**Fig 4:**
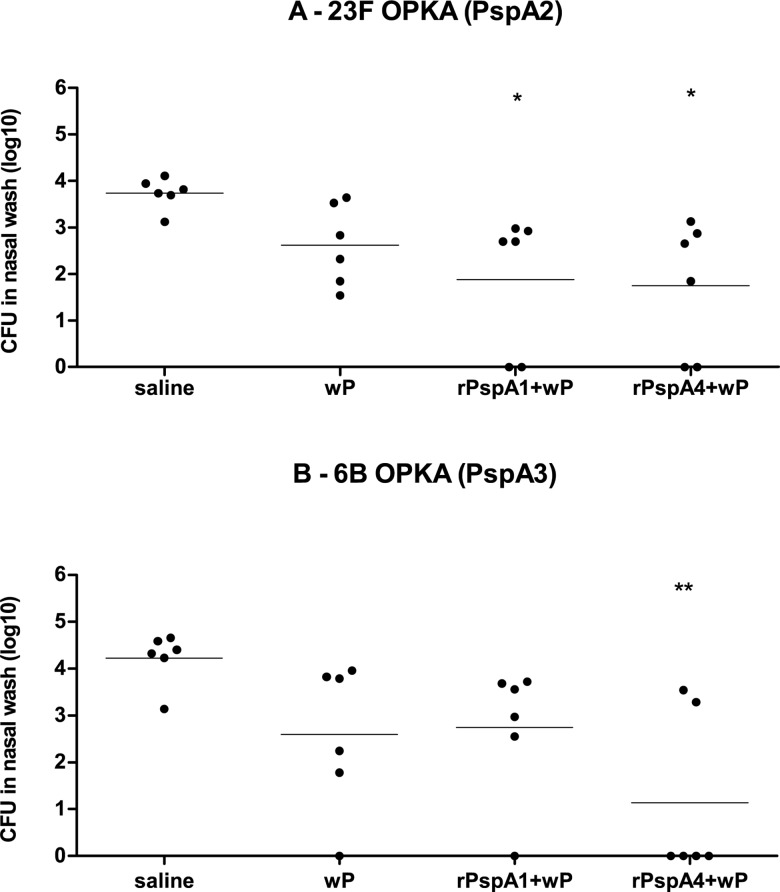
Recovery of pneumococci from the nasopharynx of mice challenged with a mixture of serotype 23F and serotype 6B strains. Mice were immunized intranasally with two doses of the indicated formulations and challenged with a mixture of serotype 23F and serotype 6B strains expressing different PspAs. Recovery of strains 23F OPKA (PspA2) (A) and 6B OPKA (PspA3) (B) in nasal washes performed 5 days after challenge is shown. * indicates statistical difference with saline (One-way ANOVA, Tukey’s Multicomparison Test- * P≤0.05; **P≤0.01).

Challenge experiments with a mixture of serotype 23F (23F OPKA—PspA2) and serotype 6B (6B OPKA—PspA3) strains were also performed. Immunization with rPspA1+wP led to statistically significant reduction in colonization by 23F OPKA (PspA2) when compared to saline. Immunization with rPspA4+wP led to statistically significant reduction in colonization with both 23F OPKA (PspA2) and 6B OPKA (PspA3) when compared to saline, but not with the adjuvant group wP (Fig 4).

**Fig 5:**
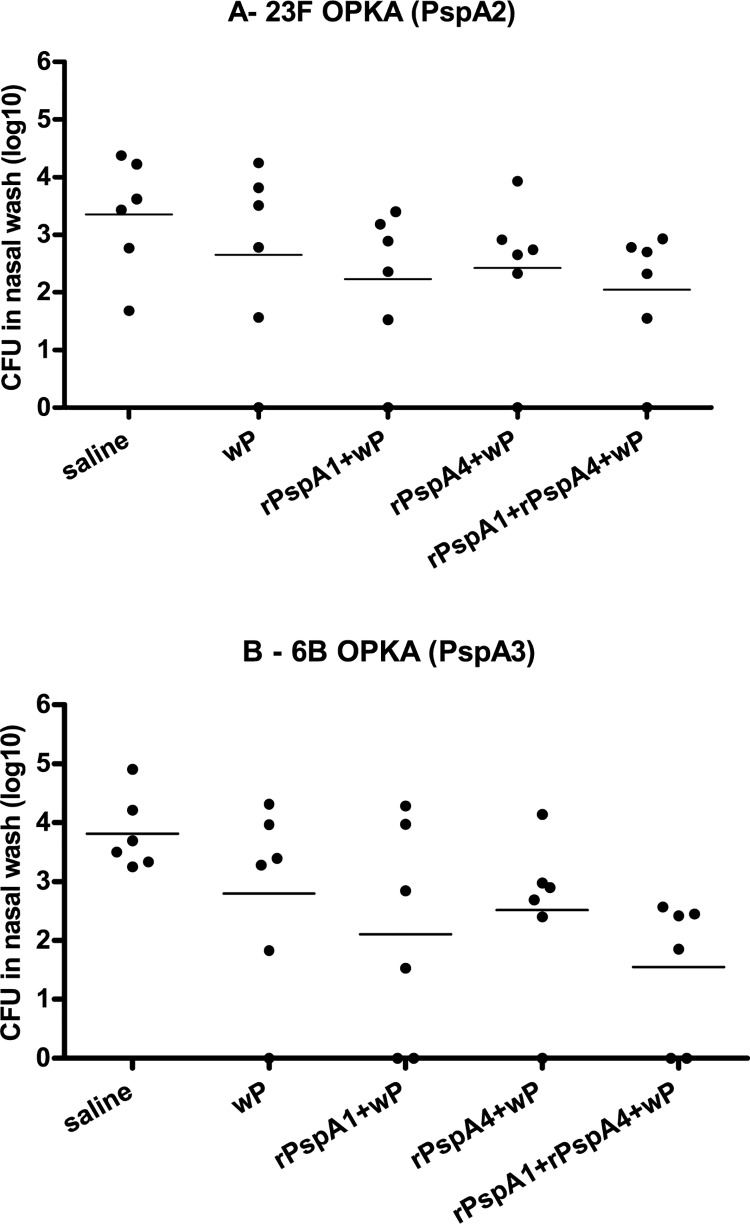
Recovery of pneumococci from the nasopharynx of mice challenged with a mixture of serotype 23F and serotype 6B strains–rPspA1 and rPspA4 mixture. Mice were immunized intranasally with two doses of the indicated formulations and challenged with a mixture of serotype 23F and serotype 6B strains expressing different PspAs. The adjuvant wP was inoculated only in the first dose. Recovery of strains 23F OPKA (PspA2) (A) and 6B OPKA (PspA3) (B) in nasal washes performed 5 days after challenge is shown.

Since all challenge experiments showed a consistent reduction in colonization by pneumococci in the control group inoculated only with the adjuvant wP, an alternative immunization protocol was tested to minimize this effect. Animals were immunized with two doses of the antigen, but wP was added only in the formulation given in first dose. Mice were then challenged with the mixture 23F OPKA (PspA2) and 6B OPKA (PspA3). Though we could observe a trend towards reduction in colonization in the groups immunized with rPspA1+wP, rPspA4+wP and rPspA1+rPspA4+wP, the differences were not statistically significant (Fig 5). Comparable levels of anti-rPspA IgG were detected with immunization with adjuvant in the two doses or only in the first dose (not shown).

### Ratio of Strains Recovered (PspA fam1:PspA fam2)

**Fig 6:**
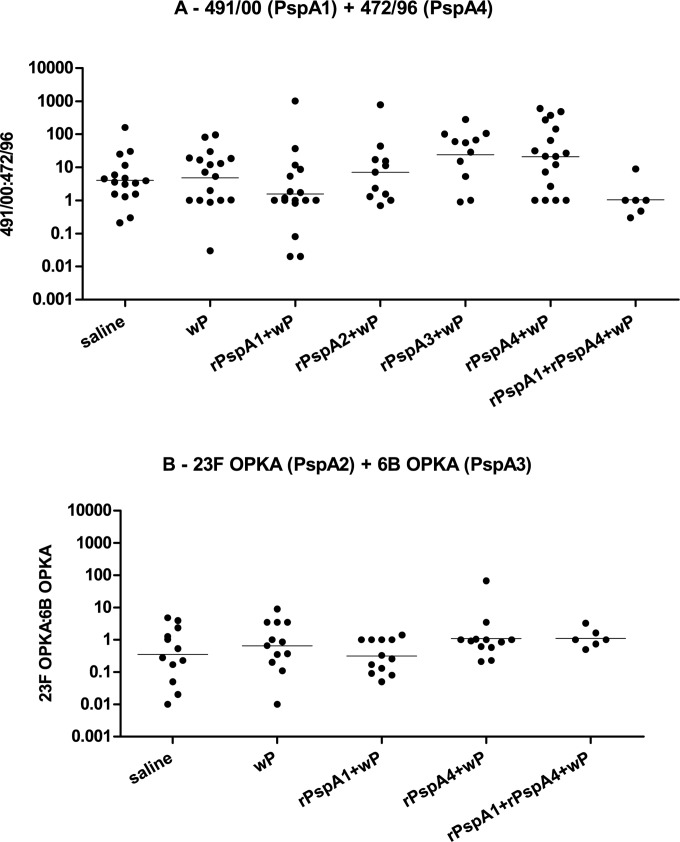
Ratio between pneumococcal strains recovered from the nasopharynx of mice challenged with a mixture of pneumococci. Ratio between strains recovered from each mouse challenged with the mixture of 491/00 (PspA1, family 1) and 472/96 (PspA4, family 2) (A) or 23F OPKA (PspA2, family1) and 6B OPKA (PspA3, family 2) (B) is shown.

The ratio between strains expressing PspA from family 1 and from family 2 recovered from each mouse challenged with the mixtures of strains St491/00 (PspA1, family 1) and St472/96 (PspA4, family 2) or 23F OPKA (PspA2, family 1) and 6B OPKA (PspA3, family 2) was analyzed. No statistical differences were observed between the ratios of the immunized groups and saline (Fig 6), which indicates that the vaccine formulations did not favor the outgrowth of one of the strains.

### Measurement of Local Immune Responses

**Fig 7:**
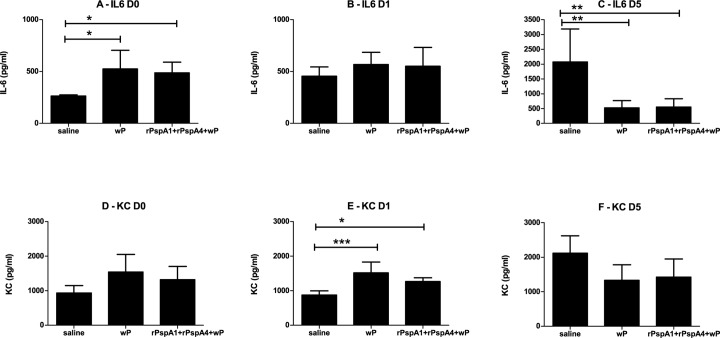
IL-6 and KC levels in nasal wash samples. NW samples were collected from mice immunized with the indicated formulations before (D0), at day 1 (D1) and at day 5 (D5) after intranasal challenge with a mixture of strains 23F OPKA (PspA2) and 6B OPKA (PspA3). IL-6 (A-C) and KC (D-F) were detected using a Luminex-based assay. * indicates statistical difference with saline (One-way ANOVA, Tukey’s Multicomparison Test—* P≤0.05; **P≤0.01, ***P≤0001). Results are representative of two independent experiments.

Local immune responses in the nasopharynx and lungs were analyzed before (D0) the intranasal challenge or at days 1 (D1) and 5 (D5) after the challenge. Mice were inoculated with saline, wP or rPspA1+rPspA4+wP and BALF and NW samples were collected. Both anti-rPspA IgG and IgA were detected in BALF and NW samples at D0 (S2 Fig). Presence of IFN-γ, IL-1-β, IL-5, IL-6, IL-10, IL17A, KC/CXCL1, MCP-1/CCL-2, MIP-2/CXCL2 and TNF-α was measured and no major differences between groups were detected in BALF samples (not shown). In NW samples, an increase in IL-6 was detected at D0 in the groups inoculated with wP and rPspA1+rPspA4+wP. At D5, control animals inoculated with saline showed higher IL-6 levels, probably as a result of higher density of colonization with pneumococci (Fig 7A–7C). An increase at D1 for KC (CXCL1) was also observed in NW samples of animals immunized with wP and rPspA1+rPspA4+wP (Fig 7D–7F).

**Fig 8:**
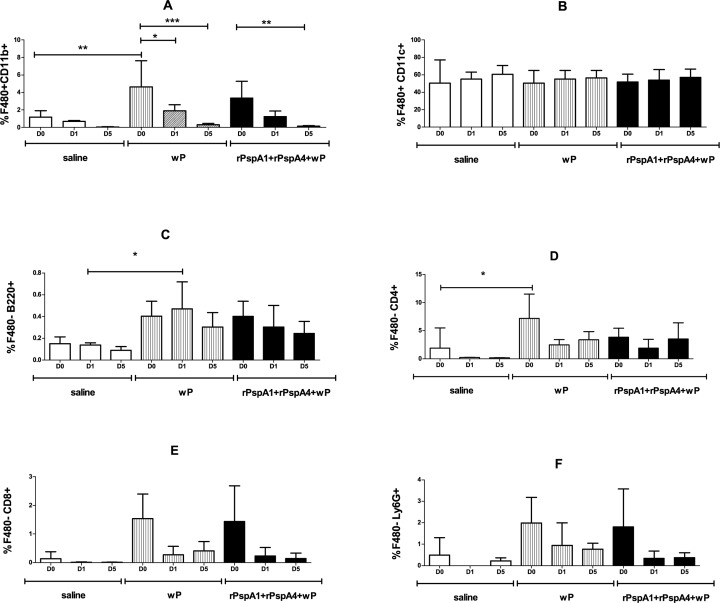
Immunophenotyping of BALF cells. BALF samples were collected from mice immunized with the indicated formulations and infiltrated cells were immunophenotyped by flow cytometry. BALF was collected before (D0), at day 1 (D1) and at day 5 (D5) after intranasal challenge with a mixture of strains 23F OPKA (PspA2) and 6B OPKA (PspA3). Percentages of F4/80^+^ CD11b^+^ (A), F4/80^+^ CD11c^+^ (B), F4/80^-^ B220^+^ (C), F4/80^-^ CD4^+^ (D), F4/80^-^ CD8^+^ (E) and F4/80^-^ Ly6G^+^ (F) are shown. Differences with saline at the same day and differences within the same group at different days are shown (One-way ANOVA, Tukey’s Multicomparison Test—* P≤0.05; **P≤0.01, ***P≤0.001). Results are representative of two independent experiments.

Immunophenotyping of cells present in BALF samples was also performed. Percentages of tissue macrophages (F4/80^+^ CD11b^+^), alveolar macrophages (F4/80^+^ CD11c^+^), B cells (F4/80^-^ B220^+^), CD4^+^ T cells (F4/80^-^ CD4^+^), CD8^+^ T cells (F4/80^-^ CD8^+^) and neutrophils (F4/80^-^ Ly6G^+^) are shown in Fig 8. No major differences were observed in the total amount of cells, since the challenge dose with pneumococci was inoculated in a small volume that does not reach the lungs (not shown). Overall, an increase in percentage of tissue macrophages, B cells, CD4^+^ T cells, CD8^+^ T cells and neutrophils was observed in the groups immunized with wP and rPspA1+rPspA4+wP at D0, with statistical significance for tissue macrophages and CD4^+^ T cells for wP compared with saline. A statistically significant increase in B cells was observed for wP at D1 when compared to saline. Furthermore, a reduction in tissue macrophages was observed in the groups immunized with wP and rPspA1+rPspA4+wP at days 1 and/or 5 after challenge when compared to D0.

## Discussion

The widespread use of PCVs led to the substitution of serotypes both in colonization and IPD. Since PspA is a variable protein, its use as vaccine antigen could lead to a phenomenon similar to serotype substitution. We have thus tested nasal immunization with rPspAs from different clades adjuvanted with wP in a model of co-colonization of mice with two strains expressing different PspAs. This model aims to mimic the common situation of simultaneous co-colonization with two or more strains in children. We have previously shown that nasal immunization with rPspA adjuvanted with wP induced protection against nasopharyngeal colonization in mice [22], but the model of co-colonization tested here should be a more stringent situation.

Nasal immunization with rPspAs+wP led to the induction of serum anti-rPspA antibodies with reactivity mostly restricted to the same family, with better results for rPspA1 and rPspA4. Induction of mucosal anti-rPspA IgG and IgA antibodies was also observed in NW and BALF samples. The first co-colonization model involved two 6B strains expressing PspA1 (family 1) or PspA4 (family 2). Mice immunized with rPspA1+wP, rPspA3+wP and rPspA4+wP showed statistically significant reduction of colonization with both strains when compared to saline. Interestingly, control mice inoculated with the adjuvant wP also showed reduction in colonization with both strains. Reduction was statistically significant for wP versus rPspA1+wP for colonization with St491/00 (PspA1) and for wP versus rPspA4+wP for colonization with St472/96 (PspA4). The immunization with the mixture rPspA1+rPspA4+wP led to a greater reduction of colonization with the two strains compared to immunization with a single antigen. The second co-colonization model involved one serotype 23F strain expressing PspA2 (family 1) and one serotype 6B strain expressing PspA3 (family 2). Once again, immunization with a mixture of rPspA1+rPspA4+wP showed a trend towards greater reduction in colonization with both strains when compared with a single antigen. Furthermore, when the ratio of recovery of the strains used in the challenge was analyzed, no major alteration was observed in mice immunized with rPspA+wP compared to saline. These results indicate that immunization with rPspA+wP seems to induce broad protection and does not favor the outgrowth of one of the strains.

The analysis of competition in colonization models with up to six pneumococcal strains from different serotypes has been previously evaluated in naïve mice. Clinical and isogenic strains expressing capsule from serotypes 6B, 14, 19A, 19F, 23F and 35B were analyzed. The experiments showed that different strains of pneumococci compete during colonization and that the serotype is a major determinant of competitive success [23]. The work also analyzed colonization with a mixture of strains of serotypes 14 and 19F in mice immunized with a non-encapsulated inactivated whole-cell pneumococcal vaccine (WCV) adjuvanted with cholera-toxin (CT). Similarly to our results, a trend towards fewer CFU recovered from mice immunized with the vaccine was found, when compared to CT alone. No differences in the ratios between strains of serotypes 14 and 19F were observed between groups.

It is clear from the reduction in colonization observed in our experiments in the group inoculated only with the adjuvant wP that the protection observed in animals inoculated with rPspA+wP formulations is due to both PspA-specific and non-specific responses. Immunization with rPspA+wP led to the induction of serum and mucosal antibodies against PspA. Antibodies against PspA can opsonize pneumococci, leading to the deposition of C3 on the bacterial surface, followed by phagocytosis [24]. Non-specific responses were observed in the groups receiving wP, characterized by an enhancement in the percentage of tissue macrophages, B cells, CD4^+^T cells, CD8^+^T cells and neutrophils in BALF samples, with statistical significance for tissue macrophages, B cells and CD4^+^T cells. A decrease in tissue macrophages was observed after challenge in the groups receiving wP, which may indicate an effector function of these cells in pneumococcal clearance in this model. An increase in IL-6 levels in NW samples before the nasal challenge with pneumococci and in KC (CXCL1) levels one day after the challenge was observed in the groups that received wP and rPspA+wP. We did not analyze NALT cells of immunized mice, but a murine model of upper respiratory tract infection has shown clonally related T cells populating both upper and lower respiratory tract [25]. Thus cells infiltrated in the lungs may reflect the T-cell population induced in the nasopharynx by the nasal immunization with rPspA+wP.

The wP vaccine used in this study is the pertussis component of the DTP (diphtheria, tetanus, pertussis) vaccine adjuvanted with alum currently given in Brazil for children through parenteral immunization. Mucosal administration of wP also seems to be a feasable strategy to prevent pertussis infection and, in fact, nasal delivery of wP has already been tested in humans. Nasal spraying of a wP vaccine without adjuvant elicited IgA responses in nasal fluid as well as IgA and IgG in serum [26]. Furthermore, vaccine-specific T-cell responses were observed, as measured by T-cell proliferation assays [27].

Memory T cells, in particular CD4^+^T cells producing IL-17A, were shown to be an important component of the acquired protective response against nasopharyngeal pneumococcal colonization in mice [28,29]. Results from an experimental human challenge model with pneumococci have also shown an increase in blood and lung IL-17A CD4^+^T memory cells after nasal pneumococcal carriage [30]. Moreover, clearance of human pneumococcal carriage associated with increased age in children was correlated with an increase in the ratio of Th17:Treg pneumococcal-specific response in NALT [31].

Both immunization with wP and lung infection with bacteria were shown to induce protective Th1 and Th17 responses against *B*. *pertussis*. Furthermore, a more severe infection in IL-17A knockout mice was associated with reduction in KC (CXCL1) production and impaired neutrophil recruitment to the lungs post challenge [32]. Our group has previously shown the induction of Th17 responses through immunization with rPspA+wP [33], but in this previous work an immunization schedule with 6 doses was used. Increased levels of IL-6 and IL-17 in BALF samples and PspA-specific secretion of IL-17 by lung and spleen cells *in vitro* were observed. We now observed a slight increase in levels of IL-17A in NW samples of mice inoculated with wP and rPspA+wP, but it did not reach statistical significance (data not shown). We did not evaluate PspA-specific secretion of IL-17A by spleen or lung cells in this current work, but the higher KC (CXCL1) levels in NW samples after challenge could reflect the induction of a Th17 response. Higher levels of the proinflammatory cytokine IL-6 in mice receiving wP or rPspA+wP would further contribute to non-specific protection against pneumococcal colonization.

A similar reduction in pneumococcal colonization has been observed using cholera-toxin B-subunit (CTB) as adjuvant for a whole-cell pneumococcal vaccine inoculated intranasally in mice [34]. This non-specific immunity against pneumococcal colonization in mice was recently associated with the activation of local innate response, involving activation of the caspase-1/11 inflammasome, mucosal T cells and macrophages [35]. An increase in IL-1β and macrophages was observed in the nasal tissue of animals inoculated with CTB. The response was characterized by the induction of IFN-γ and IL-10 and a critical role for macrophages was also described.

One limitation of this study is the evaluation of co-colonization with only two mixtures of strains and the use of only serotype 6B and 23F strains. Since there is no correlation between serotype and the expression of a specific PspA clade, the protection observed should be a good indication that immunization with rPspA+wP would be effective against co-colonization with other pneumococcal serotypes expressing different PspAs. Thus, such an immunization strategy would not favor the outgrowth of one strain, minimizing the risk of replacement. The analysis of non-vaccine serotypes and the comparison with PCVs in co-colonization models are important issues and should be further studied.

## Supporting Information

S1 Fig Binding of anti-rPspA IgG serum antibodies to the surface of intact pneumococci. (DOCX)

S2 Fig Anti-rPspA IgG and IgA in BALF and NW samples. (DOCX)

S1 Materials and Methods Materials and Methods. (DOCX)
