# Supplementary material for: Protection Elicited by Nasal Immunization with Recombinant Pneumococcal Surface Protein A (rPspA) Adjuvanted with Whole-Cell Pertussis Vaccine (wP) against Co-Colonization of Mice with Streptococcus pneumoniae
Source: PLoS One. 2017 Jan 19;12(1):e0170157. doi: 10.1371/journal.pone.0170157 (PMC5245875; doi:10.1371/journal.pone.0170157)
Supplement: S1 Fig — (DOCX) [file pone.0170157.s001.docx]

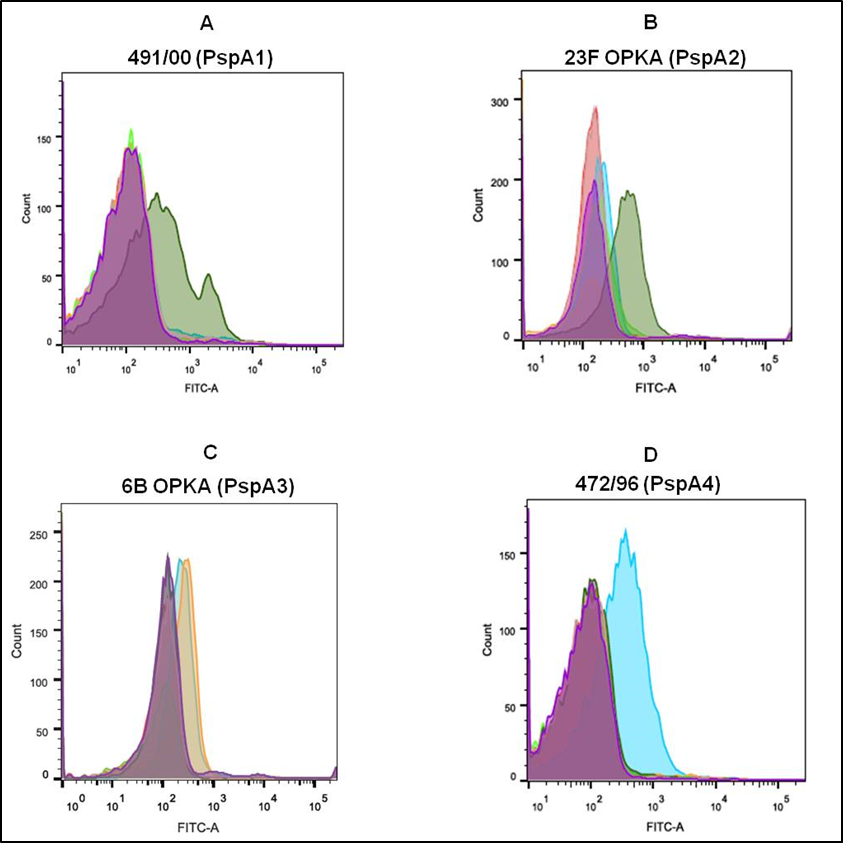


| Median: FITC-A | | | | | |
| --- | --- | --- | --- | --- | --- |
|  |  | **491/00 (PspA1)** | **23F OPKA (PspA2)** | **6B OPKA**  **(PspA3)** | **472/96 (PspA4)** |
| 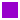 | **saline** | 57 | 132 | 107 | 34 |
| 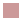 | **wP** | 61 | 144 | 94 | 37 |
| 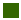 | **rPspA1+wP** | 194 | 479 | 112 | 48 |
| 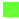 | **rPspA2+wP** | 56 | 158 | 64 | 37 |
| 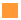 | **rPspA3+wP** | 59 | 74 | 241 | 37 |
| 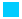 | **rPspA4+wP** | 62 | 180 | 228 | 263 |

S1 Fig. Binding of anti-rPspA IgG serum antibodies to the surface of intact pneumococci. Mice were immunized intranasally with two doses of the indicated formulations. Binding of IgG antibodies to pneumococcal strains 491/00 (PspA1) (A), 23F OPKA (PspA2) (B), 6B OPKA (PspA3) (C) and 472/96 (PspA4) was analyzed by flow cytometry using pooled serum samples. Results are shown as fluorescence intensity histograms. Results are representative of two independent experiments.
