# Supplementary material for: Protection Elicited by Nasal Immunization with Recombinant Pneumococcal Surface Protein A (rPspA) Adjuvanted with Whole-Cell Pertussis Vaccine (wP) against Co-Colonization of Mice with Streptococcus pneumoniae
Source: PLoS One. 2017 Jan 19;12(1):e0170157. doi: 10.1371/journal.pone.0170157 (PMC5245875; doi:10.1371/journal.pone.0170157)
Supplement: S2 Fig — (DOCX) [file pone.0170157.s002.docx]

S2 Fig. Anti-rPspA IgG and IgA in BALF and NW samples. Mice were immunized intranasally with two doses of the indicated formulations and anti-rPspA1 IgG (A, C), anti-rPspA4 IgG (B, D), anti-rPspA1 IgA (E, G) and anti-rPspA4 IgA (F,H) in BALF (A, B, E, F) and NW (C, D, G, H) samples were detected by ELISA. A_405_ of samples diluted 1:2 is shown. * indicates statistical difference with saline (One-way ANOVA, Tukey’s Multicomparison Test - * P≤0.5; **P≤0.01).
