# Supplementary material for: Protection Elicited by Nasal Immunization with Recombinant Pneumococcal Surface Protein A (rPspA) Adjuvanted with Whole-Cell Pertussis Vaccine (wP) against Co-Colonization of Mice with Streptococcus pneumoniae
Source: PLoS One. 2017 Jan 19;12(1):e0170157. doi: 10.1371/journal.pone.0170157 (PMC5245875; doi:10.1371/journal.pone.0170157)
Supplement: S1 Materials and Methods — (DOCX) [file pone.0170157.s003.docx]

**S1 Material and Methods**

**Antibody Binding**

Strains St491/00 (PspA1), 23F OPKA (PspA2), 6B OPKA (PspA3) and St472/96 (PspA4) were plated on blood agar overnight, then grown in THY to OD_600 nm_ 0.4-0.5 (
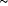
10^8^ CFU/ml) and harvested by centrifugation. Bacteria were washed, suspended in PBS and incubated with 1% of pooled sera during 30 min at 37ºC. Samples were washed once with PBS before incubation with fluorescein isothiocyanate (FITC)-conjugated anti-mouse IgG (Sigma) for 30 min on ice. Samples were fixed with 2% formaldehyde after two washing steps and stored at 4ºC. Flow cytometry analysis was conducted using FACSCalibur (BD Biosciences), and 10,000 gated events were recorded. The median of fluorescent bacteria was used to compare the groups.

**Measurement of Antibodies by Enzyme-linked Immunosorbent Assay (ELISA) in BALF and NW samples**

ELISA was carried out in plates coated with 1 μg/ml rPspA. For the detection of IgG, goat anti-mouse IgG conjugated with alkaline phosphatase (Sigma-Aldrich) was used as secondary antibody. For the detection of IgA, goat anti-mouse IgA (Southern Biotech) and rabbit anti-goat IgG conjugated with alkaline phosphatase (Sigma) was used. A_405_ of samples diluted 1:2 is shown.
